# The role of mood in shaping reactions to smoking cessation messages among adults who smoke: a multimodal investigation

**DOI:** 10.1186/s12889-024-20140-5

**Published:** 2024-10-18

**Authors:** Elise M. Stevens, Donghee N. Lee, Hannah Stevens, Rajani S. Sadasivam

**Affiliations:** 1 Department of Population and Quantitative Health Sciences, Division of Preventive and Behavioral Medicine, UMass Chan Medical School, Worcester, MA USA; 2 Department of Population and Quantitative Health Sciences, Division of Health Informatics and Implementation Science, UMass Chan Medical School, Worcester, MA USA

**Keywords:** Communications, Smoking Cessation messages, Mood

## Abstract

**Introduction:**

Mood-tailored communications may help increase the effectiveness of smoking cessation messaging interventions. We used both self-report and psychophysiological measures to test the impact of mood on responses to cessation messages in adults who smoke.

**Methods:**

In a two-part (crowdsourcing and psychophysiological studies) study, the impact of 30 smoking cessation messages comprised of five themes (i.e., financial, health, quality-of-life, challenges in quitting, motivation to quit) were tested. In a crowdsourcing study, participants (*N* = 600) were randomly placed into one of three mood induction tasks (i.e., positive, negative, neutral), and then viewed the smoking cessation messages. After each message, they were asked to self-report their motivation to quit, message receptivity, and the perceived relevance of the messages. In an in-lab, psychophysiological study, participants (*N* = 42) completed the same tasks as the crowdsourcing participants but were monitored for heart rate, skin conductance, and eye-tracking while viewing the cessation messages. Using a multi-attribute decision-making model (MADM) using outcomes from both studies, messages were ranked for each mood state.

**Results:**

The top messages for participants in the positive mood condition included the challenges in quitting, financial costs/rewards, and motivations to quit themes. The top messages for participants assigned to the negative mood condition included the challenges in quitting, quality-of-life, and financial costs/rewards themes. For participants in the neutral mood condition, messages in the challenges in quitting and quality of life themes performed best.

**Conclusions:**

Variations in the preferences of messages and themes by mood condition suggest that mood-tailored communication may increase the effectiveness of smoking cessation messages.

## Introduction

Smoking is the leading cause of preventable death in the United States (U.S.) [1]. Smoking is linked to 12 cancer types [2] and can be attributed to 36% of all cancer deaths [1, 3, 4]. Innovative and scalable interventions are needed to increase cessation rates [5]. Computer-tailored health communication (CTHC), which uses computer programs to select the best messages for an individual, can effectively support smoking cessation efforts [6–13]. However, more research is needed to understand which messages will work best for individual differences so interventions are tailored accordingly.

Previous research has employed CTHC in smoking cessation studies on its own (e.g., texting) or as part of an intervention, and several real-world health messaging programs have integrated CTHS [14–16]. CTHC programs usually select messages based on a participant’s demographic characteristics (e.g., age, gender) or upon key expert-identified variables (e.g., readiness to quit smoking) [17]. Research has also explored the use of mobile phones to collect contextual factors (e.g., location or current participant stress) to develop just-in-time CTHC interventions. Mood, a key contextual factor often incorporated in commercially used algorithms, has yet to be incorporated into CTHC and could be the next CTHC advancement. For example, decades of research on consumer behavior have shown current mood to be one of the biggest influences on purchasing behavior [18–20], and many innovative companies, such as Apple, are developing mood-tailored messaging (selecting the best messages for a specific mood) to increase the content’s influence on the customer [21–23]. Furthermore, limited academic research suggests that pre-existing mood, may in fact, impact the effectiveness of health communication [24, 25]. For example, one study found that individuals in a positive mood state who smoked were able to better systematically process information from a message about smoking cessation (i.e., identify high- and low-quality arguments) than those in a negative mood state [25]. In another study examining pre-existing mood on responses to self-reported health message evaluation, results showed that mood, both positive and negative, impacted responses to the type of messages viewed (i.e., prevention vs. detection) [24]. Additionally, a pilot study focused on individuals who smoke (*n* = 14), asked participants about their moods followed by another text message assessing their interest in connecting with a tobacco treatment specialist via text messaging twice a week over the course of five weeks. Participants were more likely (42%) to express interest in connecting with the tobacco treatment specialist if they reported a neutral mood and less likely when in a positive or negative mood. Additionally, in our previous work, we found that positive mood (vs. negative mood) increased self-reported motivations to quit after viewing smoking cessation messages with financial, health, quality of life, and challenges in quitting themes [26]. These studies and a limited number of laboratory studies suggest that mood may affect how adults who smoke process cessation messages and behavior, such as following a referral to a cessation resource [25, 27, 28]. However, no research has specifically examined how pre-existing mood impacts responses to CTHC messages with differing themes about smoking cessation using multiple methodologies, especially beyond self-report.

Mood as information theory posits that mood can impact how a message is processed. Specifically, when a person is in a positive mood, which conveys feelings of safety and security, they are more inclined to engage in superficial processing of information, as their mood diminishes the perception of potential threats [29]. Research also suggests that individuals in a negative mood may use more effortful processing [29]. Likewise, mood management theory suggests that if one is in a positive mood state, they are likely to want to continue that state, not processing messages too elaboratively to decrease the chances of their mood turning negative [27, 30]. Thus, using these theoretical frameworks, we examined how mood may impact responses to CTHC smoking cessation messages.

Mood is a temporary but generalized affective state that forms from an individual’s emotions (e.g., feeling happy or depressed) and has the ability to influence one’s cognitive and behavioral responses [31–35]. Unlike emotions, which are shorter lived and more intense [36], mood is typically described as positive, negative, or neutral. One’s mood has been shown to impact message judgement [37], memory of a message [38], and message recall [39]. Furthermore, in studies that induced mood with stimuli preceding the message, results showed that, based on mood, individuals had different evaluations of the message [40] and recalled that information later on differently [41]. Thus, we should expect that mood may also impact how individuals respond to messages about smoking cessation intended for a digital intervention. This is especially important as individuals may receive these messages when in differing mood states and CTHC allows for mood tailoring. However, knowing which messages are best for which mood state is an underexplored research question.

For the current study, we used previously tested and highly rated smoking cessation messages from a CTHC study to examine how smoking cessation messages affect responses among adults who smoke when in particular mood states (i.e., positive, negative, neutral), innovatively employing a blend of crowdsourcing and psychophysiological data [42–44]. Psychophysiological measurement objectively measures heart rate, skin conductance, and eye-tracking to obtain real-time responses during message consumption to complement self-report data [45, 46]. Heart rate measures cognitive resources allocated to message processing [46]. Skin conductance measures arousal levels during message exposure [46]. Eye-tracking measures visual attention to aspects of the message. Collecting psychophysiological data alongside self-report data allows researchers to reduce self-report bias [46], making it the optimal strategy for formatively testing messaging.

Multi-attribute decision-making models (MADM) allow researchers to integrate data from multiple inputs (i.e., self-report and psychophysiology) to determine rankings of messages that performed best [47]. Using the MADM framework [47], a decision matrix is created in which each row (e.g., message) represents a message and each column represents a construct collected via the crowdsourcing study and the psychophysiological study. The rank of each construct is averaged for each message to create a total rank for each message by measure type [47]. Then, all messages are ranked overall, resulting in an overall top message. MADM provides a practical, transparent framework for considering data across multiple inputs, determining the relative importance of each attribute, and simultaneously evaluating detail at the attribute level and in a summary measure across all attributes using aggregate data [48]. MADM has been widely used in business [49], economics [50], health care [51], and most recently by our team in health communication science [47]. 

The main aims of this two-part study were to determine how distinct mood states (i.e., positive, negative, neutral) impact self-reported relevancy, receptivity, and motivation to quit and how they respond to CTHC messages in real-time using psychophysiology. The results with only the crowdsourcing data, using significance testing, have been published elsewhere [26]. Briefly, those results showed that participants in the positive mood condition had greater motivation to quit after seeing the messages than those in the negative mood condition, especially after seeing the financial, health, quality of life, and challenges in quitting theme categories [26]. Thus, the current study first examined psychophysiological responses (i.e., heart rate, skin conductance) to the messages using significance testing. Then, the data from the crowdsourcing study and psychophysiology study were integrated using a MADM to rank each message for each mood state to determine the most effective messages for adults who smoke depending on their mood state.

## Methods

### Crowdsourcing study

#### Participants

In January 2022, participants were recruited from a crowdsourcing platform (Prolific.com). Prolific prescreens all of its participants and allows researchers to choose from various inclusion criteria. Inclusion criteria included: (1) being 18 years and/or older, (2) currently smoking cigarettes (smoking at least 5 cigarettes per day and having smoked that amount for at least one year), and (3) currently residing in the U.S.

#### Procedures

Participants (*N* = 600) who met the inclusion criteria completed an online consent form after being directed to a Qualtrics survey. Then, participants were randomly assigned to one of three mood induction conditions: (1) positive (*N* = 200), (2) negative (*N* = 200), or (3) neutral (*N* = 200). Participants in each condition viewed one picture that induced the mood in which they were randomly assigned and then viewed one smoking cessation message. After viewing the cessation message, participants were asked about their motivation to quit, message receptivity, and their perceived relevance of the message. This process was repeated 29 more times. In other words, participants saw a mood picture, then saw a smoking cessation message, then answered questions about that message. They did this process 30 times. All participants in all mood conditions saw the same 30 smoking cessation messages in a random order. Mood induction pictures were selected from the International Affective Picture System (IAPS) [52], which is a database of colored pictures that have been rated and validated to be normative emotional stimuli for experimental studies. Following Coan’s (2007) guidelines [53], we selected pictures depicting pleasant (positive), neutral, and unpleasant (negative) activities. For the positive mood condition, pictures were selected that elicited happy, loving, and nurturing emotions. For the negative mood condition, pictures were selected that elicited sad, angry, afraid, anxious emotions. However, pictures with graphic visuals that evoked erotica (sexual or romantic) or extreme disgust, such as mutilation, or contamination were unselected. In alignment with previous studies, the positive pictures included animals, babies, children, and families. Neutral mood pictures included objects or adults with neutral facial expressions. Negative mood pictures included guns, accidents, and aggressive animals [54, 55]. After each of the first three pictures, participants were asked to rate their mood as a manipulation check. After the message viewing task, participants were asked to complete a series of demographic items. Participants were compensated $3.97 upon completion via Prolific. The entire survey lasted 20–25 min. All procedures were approved by the University of Massachusetts Chan Medical School’s Institutional Review Board.

#### Message selection

The messages in this study were included as part of a CTHC system in a prior trial. These messages were developed through an iterative group review process guided by theoretical frameworks and existing smoking cessation guidelines as well as written by peer individuals who smoke [56]. In our randomized trial with 900 individuals who smoke, compared to a website control; those who received the messages had higher of quitting smoking than comparison participants [Odds Ratio 1.69 (95% CI 1.03–2.8)] [57, 58]. Then, we had 846 individuals who smoke rate our messages [59]. The messages that were rated as top messages by these individuals were used in the current study [17]. There were six messages in each theme category: (1) motivation to quit, (2) challenges in quitting, (3) quality-of-life, (4) health harms, and (5) financial costs/rewards. Each message was also assigned a secondary category as themes crossed categories.

#### Measures

##### Motivation to quit

Motivation to quit smoking was measured using the item “How motivated are you to quit smoking?” from 1 (Not at all motivated) to 10 (Very motivated) [60]. Scores were averaged across each message, giving each message its own score for each mood condition.

##### Message receptivity

Message receptivity was measured by asking participants the extent to which they agreed the message was appealing, spoke to them, said something important to them, was convincing, would motivate persons to prevent smoking, was confusing, promoted behaviors that are difficult, they did not like the messages, and contradicts what they know about smoking. Items were on a scale from 1 (Strongly disagree) to 5 (Strongly agree) [61]. The last four items were reverse coded, and then the mean was taken of the items. Next, scores were averaged across each message, giving each message its own score for each mood condition.

##### Perceived relevance of the message

Perceived relevance was measured by asking participants how much they felt the message was relevant to their life, grasped their attention, and said something important to them on a scale from 1 (Strongly disagree) to 7 (Strongly agree) [62]. Items were averaged. Then, scores were averaged across each message, giving each message its own score for each mood condition.

### Psychophysiological study

#### Participants

Participants (*N* = 42) were recruited through social media, mass emails, and past participant databases at a large northeastern university. Inclusion criteria included: (1) currently smoking (smoking daily or most days) and (2) had smoked at least 100 cigarettes in their lifetime.

#### Procedures

After meeting the inclusion criteria, participants were invited into the lab for a one-hour session. After obtaining informed consent, participants were seated at a computer screen where procedures were identical to those in the crowdsourcing study, except participants were monitored for heart rate, skin conductance via sensors on their fingers, and eye-tracking via a camera on the computer screen (N_positive_ = 14; N_negative_ = 15; N_neutral_ = 13), and participants reported their mood state after each emotion induction picture. Participants viewed the messages for 15 s before the computer automatically moved on to the next screen. Upon completion, participants were compensated $50. All procedures were approved by the University of Massachusetts Chan Medical School’s Institutional Review Board.

#### Measures

##### Heart rate

Participants’ heart rate while viewing the messages was measured to determine the cognitive resources allocated to message processing [46]. Three sensors were attached to participants’ clean fingertips on the left hand using a Shimmer EXG module. Raw data were sampled at 512 Hz. Heart rate change scores were computed using beats per minute (BPM) by subtracting the first second of the message from scores from the number of seconds each participant was exposed to the message. Then, scores were averaged across each message, giving each message its own score for each mood state. Lower values indicated greater resources allocated to message processing. In other words, deceleration indicates more resources allocated [46].

##### Skin conductance

Skin conductance during message viewing measured participant arousal levels. The Shimmer EXG module recorded this measure. Each message was assigned a score for the mean amplitude of “peaks” from all participants. A “peak” indicates how high a participant’s biological arousal was when viewing the message [46]. For significance testing, change scores were computed by subtracting the first second of the message from scores from the number of seconds each participant was exposed to the message.

##### Visual attention

Visual attention was measured using eye-tracking. Overall dwell time in milliseconds was recorded using an eye-tracker on the base of the computer screen (EyeTech VT3). Each message received a mean dwell time computed from all participants’ dwell time while viewing within each mood condition.

#### Data integration and analysis

First, the heart rate and skin conductance change scores were submitted to a 5 (theme: motivation to quit, challenges in quitting, quality-of-life, health harms, financial costs/rewards × 6 (message per theme) × 14 (time in seconds) repeated measures ANOVA. Due to the number of messages and the number of participants, significance testing was done to examine which theme performed best for each mood state. Since each message was on the screen for the same amount of time, significance tests were not run for dwell time (eye-tracking).

Next, self-report scores from the crowdsourcing study were averaged across participants in each mood condition for each message for each measure. Psychophysiological scores were also averaged across participants in each mood condition for each message for each measure. Then, scores were ranked (1 = most effective; 30 = least effective) for each message in each mood condition using a MADM. Higher scores of the self-report, skin conductance, and eye-tracking measures were ranked as more effective. Lower scores of heart rate were ranked as more effective [46]. See Tables 1 and 2, and 3 for MADM rankings for each mood state. See Table 4 for exact wording of the messages.

**Table 1:** Multi-attribute decision-making model for positive Mood State

Message #	Theme	Motivation to Quit	Message Receptivity	Perceived Relevance	Overall Self-report ranking	Skin	Heart Rate	Eye-Tracking Overall	Psychophysiology Overall	Overall Ranking
A9	Challenges of quitting	6.73 (3.04)	3.97 (0.80)	5.06 (1.96)	1	0.1639	2.09	14997.83	4	1
A30	Financial	5.29 (2.89)	3.47 (0.83)	3.91 (1.98)	6	0.089578	0.91	14997.71	1	2
A1	Motivations to quit	6.56 (3.03)	3.92 (0.89)	5.02 (2.05)	4	0.090214	-0.28	14995.72	3	2
A27	Financial	5.6 (2.92)	3.49 (0.82)	3.8 (1.96)	9	0.110244	1.82	14999.36	2	4
A7	Challenges of quitting	6.53 (3.08)	3.98 (0.80)	4.98 (2.00)	2	0.057643	0.21	14995.83	10	5
A4	Motivations to quit	5.44 (3.10)	3.47 (0.83)	3.71 (1.97)	6	0.08831	1.66	14996.51	7	6
A11	Challenges of quitting	6.2 (2.92)	3.78 (0.80)	4.41 (1.94)	8	0.072286	1.37	14996.67	5	6
A6	Motivations to quit	5.91 (3.09)	3.73 (0.86)	4.52 (2.04)	4	0.033314	1.56	14996.8	12	8
A18	Quality of life	6.13 (2.95)	3.81 (0.81)	4.46 (2.00)	5	0.1812	1.93	14995.29	11	8
A20	Health	5.82 (2.99)	3.64 (0.91)	4.46 (2.08)	9	0.09262	2.47	14998.44	7	8
A10	Challenges of quitting	6.79 (2.92)	4.02 (0.79)	4.97 (1.90)	1	0.06194	2.85	14998.27	15	8
A3	Motivations to quit	6.12 (2.98)	3.74 (0.82)	4.5 (1.90)	4	0.033314	1.45	14996.49	13	12
A28	Financial	5.52 (2.79)	3.52 (0.76)	3.94 (1.98)	8	0.0543	1.4	14997.4	9	12
A15	Quality of life	5.83 (2.99)	3.73 (0.85)	4.46 (2.03)	13	0.0648	1.31	14996.53	6	14
A26	Financial	6.17 (3.06)	3.78 (0.81)	4.34 (2.01)	5	0.0219	1.15	14996.09	15	15
A2	Motivations to quit	6.38 (2.94)	3.89 (0.80)	4.85 (1.89)	4	0.0669	1.87	14995.14	18	16
A5	Motivations to quit	6.05 (3.00)	3.7 (0.84)	4.39 (1.97)	5	0.078883	2.54	14995.35	20	17
A29	Financial	5.86 (3.05)	3.77 (0.82)	4.56 (2.14)	5	0.021657	0.93	14994.26	21	18
A23	Health	5.73 (3.00)	3.55 (0.81)	4.09 (1.89)	10	0.127386	4.12	14995.78	17	19
A16	Quality of life	6.61 (2.99)	4 (0.77)	5.01 (1.83)	2	0.044289	1.98	14994.93	25	19
A21	Health	5.53 (2.99)	3.46 (0.87)	3.88 (1.99)	14	0.065038	2.05	14996.38	13	19
A22	Health	5.64 (3.08)	3.59 (0.87)	4.24 (2.00)	10	0.078483	2	14995.22	18	22
A8	Challenges of quitting	6.38 (2.90)	3.92 (0.77)	4.86 (1.98)	2	0.03024	2.61	14996.06	28	23
A25	Financial	5.8 (3.03)	3.66 (0.83)	4.13 (2.10)	9	0.05086	2.39	14995.39	22	24
A14	Quality of life	6.1 (3.01)	3.77 (0.83)	4.44 (2.04)	8	0.018563	0.46	14992.73	23	24
A13	Quality of life	6.12 (3.07)	3.85 (0.83)	4.49 (2.03)	6	0.03245	1.48	14993.73	26	26
A17	Quality of life	6.01 (3.08)	3.74 (0.80)	4.32 (2.05)	11	0.034986	2.15	14995.64	23	27
A24	Health	5.35 (3.01)	3.44 (0.87)	3.9 (2.05)	12	0.04966	2.56	14995.26	29	28
A19	Health	5.43 (2.96)	3.36 (0.85)	3.98 (1.96)	17	0.052571	4.27	14994.93	30	29
A12	Challenges of quitting	5.29 (3.19)	3.15 (0.96)	3.41 (2.03)	27	0.0197	2.1	14995.89	26	30

Note. Overall ranking for self-report was calculated by adding the means of motivation to quit, message receptivity, and perceived relevance. Then, scores were ranked with higher scores ranking better. Overall psychophysiological ranking was calculated by taking the rank score for each measure (heart rate, skin conductance, overall eye-tracking) within its measure category and summing it with each of the other psychophysiological rankings. Overall ranking was computed by taking the mean of the overall self-report ranking and psychophysiological ranking. Then, based on the mean, each message was assigned an overall ranking. Self-report means are shown for each message and standard deviations are in parentheses

**Table 2:** Multi-attribute decision-making model for negative Mood State

Message #	Theme	Motivation to Quit	Message Receptivity	Perceived Relevance	Overall Self-report ranking	Skin	Heart Rate	Eye-Tracking Overall	Psychophysiology Overall	Overall Ranking
A10	Challenges of quitting	5.93 (2.98)	3.85 (0.83)	4.84 (2.02)	1	0.07015	-0.44	14997.42	2	1
A16	Quality of life	5.93 (2.93)	3.8 (0.84)	4.72 (1.96)	4	0.0468	0.11	14996.48	8	2
A26	Financial	5.42 (3.22)	3.62 (0.92)	4.2 (2.23)	16	0.05775	-0.71	14998.43	1	3
A8	Challenges of quitting	5.81 (3.10)	3.75 (0.89)	4.69 (2.05)	6	0.0772	2.35	14996.99	11	3
A18	Quality of life	5.35 (3.00)	3.65 (0.89)	4.32 (2.05)	15	0.0731	-0.28	14997.09	3	5
A14	Quality of life	5.44 (2.98)	3.58 (0.93)	4.34 (2.10)	13	0.0383	0.422	14999.31	6	6
A9	Challenges of quitting	5.92 (3.10)	3.81 (0.81)	4.81 (2.02)	2	0.05445	1.03	14995.66	18	7
A3	Motivations to quit	5.37 (3.00)	3.58 (0.86)	4.26 (2.08)	17	0.0683	0.76	15012.82	4	8
A2	Motivations to quit	5.59 (3.19)	3.78 (0.91)	4.71 (2.09)	7	0.0387	0.86	14997.03	14	8
A1	Motivations to quit	5.73 (3.13)	3.76 (0.91)	4.84 (2.04)	5	0.021775	1.05	14998.63	17	10
A20	Health	5.47 (3.06)	3.58 (0.85)	4.41 (1.99)	11	0.05155	2.44	14998.87	12	11
A29	Financial	5.42 (3.06)	3.71 (0.92)	4.56 (2.24)	8	0.03155	0.54	14996.24	18	12
A7	Challenges of quitting	5.77 (3.03)	3.82 (0.85)	4.88 (2.10)	3	0.04605	3.19	14996.86	24	13
A5	Motivations to quit	5.45 (3.08)	3.65 (0.86)	4.38 (2.11)	10	0	0.04	14996.5	18	14
A13	Quality of life	5.36 (2.98)	3.68 (0.84)	4.5 (2.08)	9	0.0222	2.43	14997.72	22	15
A28	Financial	4.93 (2.82)	3.46 (0.86)	4.01 (2.06)	23	0.09714	0.76	14995.14	9	16
A4	Motivations to quit	4.8 (3.02)	3.38 (0.90)	3.7 (2.14)	28	0.04646	0.37	14998.25	5	17
A30	Financial	4.83 (2.85)	3.47 (0.87)	3.94 (2.08)	24	0.0216	0.39	14998.05	9	17
A17	Quality of life	5.43 (3.11)	3.62 (0.90)	4.36 (2.17)	12	0.026325	0.61	14995.85	21	17
A27	Financial	5.07 (2.97)	3.46 (0.85)	3.89 (2.12)	22	0.243267	1.83	14996.07	12	20
A12	Challenges of quitting	4.46 (2.98)	3.00 (1.01)	3.12 (2.12)	30	0.0358	-1.09	14997.12	6	21
A6	Motivations to quit	5.21 (3.07)	3.58 (0.88)	4.33 (2.14)	20	0.125425	0.87	14994.76	16	21
A11	Challenges of quitting	5.49 (2.93)	3.61 (0.79)	4.23 (1.96)	14	0.019567	2.3	14996.69	28	23
A24	Health	4.6 (3.02)	3.31 (0.88)	3.95 (2.08)	29	0.071667	0.63	14995.04	14	24
A22	Health	5.27 (2.98)	3.55 (0.87)	4.33 (2.15)	19	0.058033	2.07	14994.22	26	25
A15	Quality of life	5.27 (3.07)	3.62 (0.87)	4.31 (2.10)	18	0.03088	1.49	14994.99	28	26
A23	Health	4.85 (3.00)	3.36 (0.87)	3.79 (2.16)	26	0.0331	2.09	14996.76	22	27
A21	Health	4.92 (2.98)	3.35 (0.86)	3.86 (2.07)	25	0.0523	3.08	14996.25	24	28
A25	Financial	5.22 (3.07)	3.52 (0.95)	4.18 (2.11)	21	0.0212	1.41	14995.27	30	29
A19	Health	4.62 (3.01)	3.29 (0.88)	3.99 (2.03)	27	0.0215	0.33	14993.84	27	30

Note. Overall ranking for self-report was calculated by adding the means of motivation to quit, message receptivity, and perceived relevance. Then, scores were ranked with higher scores ranking better. Overall psychophysiological ranking was calculated by taking the rank score for each measure (heart rate, skin conductance, overall eye-tracking) within its measure category and summing it with each of the other psychophysiological rankings. Overall ranking was computed by taking the mean of the overall self-report ranking and psychophysiological ranking. Then, based on the mean, each message was assigned an overall ranking. Self-report means are shown for each message and standard deviations are in parentheses

**Table 3:** Multi-attribute decision-making model for Neutral Mood State

Message #	Theme	Motivation to Quit	Message Receptivity	Perceived Relevance	Overall Self-report ranking	Skin	Heart Rate	Eye-Tracking Overall	Psychophysiology Overall	Overall Ranking
A10	Challenges of quitting	6.26 (2.84)	3.90 (0.75)	4.95 (1.86)	1	0.05802	0.39	14998.17	5	1
A15	Quality of life	5.78 (2.95)	3.68 (0.81)	4.51 (2.00)	10	0.056667	-0.84	14997.72	2	2
A8	Challenges of quitting	5.89 (2.96)	3.78 (0.82)	4.67 (1.98)	7	0.11745	1.25	14998.72	5	2
A11	Challenges of quitting	5.68 (2.81)	3.73 (0.77)	4.41 (1.91)	12	0.1532	-0.37	14996.65	1	4
A9	Challenges of quitting	6.02 (2.87)	3.83 (0.77)	4.87 (1.84)	5	0.091533	0.09	14995.55	9	5
A16	Quality of life	6.14 (2.90)	3.89 (0.78)	4.85 (1.95)	3	0.0411	-0.2	14,996	13	6
A6	Motivations to quit	5.51 (2.86)	3.69 (0.79)	4.49 (2.00)	13	0.047475	-0.88	14998.45	3	6
A18	Quality of life	5.62 (2.79)	3.64 (0.82)	4.42 (1.91)	14	0.0816	0.97	14996.74	8	8
A5	Motivations to quit	5.55 (2.95)	3.60 (0.82)	4.29 (2.00)	17	0.166267	-0.65	14994.75	7	9
A13	Quality of life	5.82 (2.91)	3.79 (0.83)	4.42 (1.98)	9	0.092125	6.14	14996.61	16	10
A14	Quality of life	5.78 (2.91)	3.76 (0.82)	4.65 (2.01)	8	0.018933	0.23	14996.65	17	10
A1	Motivations to quit	6.08 (3.03)	3.89 (0.79)	4.86 (2.00)	4	0.0331	2.72	15000.73	22	12
A20	Health	5.55 (2.86)	3.58 (0.85)	4.45 (1.95)	15	0.0704	1.54	14997.69	11	12
A7	Challenges of quitting	6.05 (2.94)	3.91 (0.83)	5.06 (1.92)	2	0.067	1.44	14995.24	25	14
A26	Financial	5.53 (2.97)	3.64 (0.87)	4.18 (2.15)	19	0.07122	1.13	14996.29	12	15
A24	Health	4.88 (2.88)	3.33 (0.84)	3.65 (1.99)	28	0.289575	0.97	14996.96	4	16
A2	Motivations to quit	5.93 (2.97)	3.88 (0.83)	4.8 (2.01)	6	0.0445	1.85	14995.34	29	17
A22	Health	5.29 (2.70)	3.49 (0.84)	4.26 (1.92)	20	0.02325	-0.07	14996.19	17	18
A3	Motivations to quit	5.45 (2.95)	3.66 (0.82)	4.42 (2.00)	16	0.088575	0.97	14994.29	21	18
A21	Health	5 (2.84)	3.42 (0.81)	3.81 (2.00)	25	0.0535	0.77	14996.06	14	20
A4	Motivations to quit	4.77 (2.83)	3.38 (0.80)	3.5 (1.93)	29	0.03905	0.96	15001.36	10	20
A17	Quality of life	5.44 (2.99)	3.68 (0.81)	4.3 (2.01)	18	0.048675	0.91	14995.47	22	22
A28	Financial	5.07 (2.74)	3.43 (0.77)	3.87 (2.02)	23	0.04024	0.48	14996.19	17	22
A29	Financial	5.54 (2.79)	3.78 (0.83)	4.56 (2.00)	11	0.0398	1.43	14994.86	30	24
A19	Health	4.83 (2.87)	3.27 (0.78)	3.89 (1.91)	27	0.0523	-0.14	14995.23	15	25
A23	Health	5.03 (2.74)	3.42 (0.79)	3.88 (1.90)	24	0.119533	1.15	14994.64	20	26
A25	Financial	5.17 (2.85)	3.56 (0.88)	4.04 (2.07)	21	0.0221	0.28	14995.9	24	27
A27	Financial	5.08 (2.90)	3.51 (0.85)	3.92 (2.00)	22	0.03416	3.06	14997.13	26	28
A30	Financial	4.97 (2.79)	3.42 (0.79)	3.73 (1.91)	26	0.022083	1.64	14996.74	28	29
A12	Challenges of quitting	4.67 (2.97)	3.11 (0.95)	3.17 (2.06)	30	0.0498	6.69	14996.53	27	30

Note. Overall ranking for self-report was calculated by adding the means of motivation to quit, message receptivity, and perceived relevance. Then, scores were ranked with higher scores ranking better. Overall psychophysiological ranking was calculated by taking the rank score for each measure (heart rate, skin conductance, overall eye-tracking) within its measure category and summing it with each of the other psychophysiological rankings. Overall ranking was computed by taking the mean of the overall self-report ranking and psychophysiological ranking. Then, based on the mean, each message was assigned an overall ranking. Self-report means are shown for each message and standard deviations are in parentheses

**Table 4:** Message texts and themes

Message ID	Message Text	Theme
A1	Brandon, a current smoker, said that to get focused on quitting you should get psyched about a vacation, and the money you spend on cigs could go to that.	5
A2	Tina, a young smoker, said It’s important for you to think about longevity. Many people want to see their children grow	5
A3	Along with all the other health benefits of quitting, you will also notice improvement in the appearance of your hands and nails once you quit.	5
A4	Make a list of why you want to quit smoking. Each day, use the list as a reminder of your reasons for wanting to quit.	5
A5	Need another reason to quit smoking? Quitting may help you feel better about yourself and will help keep your children healthier.	5
A6	Are you worried about how smoking affects your family and friends? Try to avoid smoking around your loved ones.	5
A7	Did you know smoking can influence your mood? If you feel lonely or depressed while quitting, talk with your doctor. There is treatment to help.	4
A8	There will be challenges to quitting, especially during the first few weeks. Make a list of things you can do, like exercise, to help with these challenges.	4
A9	Realize the first 48 h after quitting is the most difficult time. Make a plan to handle it. It gets better!	4
A10	Feelings of stress are normal when quitting smoking. You are not alone! Talk with your doctor or a friend about ways to reduce stress before your quit date.	4
A11	The worst withdrawal symptoms will occur in the first week after quitting, but by one month, most symptoms are gone.	4
A12	People often smoke when they are stressed, to relax, after eating, and while driving. What triggers your smoking?	4
A13	Quitting will have a positive impact on your physical ability and will help you perform better in your life. You can do this. Your doctor is ready to help.	3
A14	Smoking depletes the skin’s natural glow and creates fine lines. Quitting smoking can help reverse the harm that smoking has done to your skin.	3
A15	No matter how many years you have been smoking, quitting can increase your life span and give you a better quality of life.	3
A16	The smell of smoke gets into your clothes, your car, your home, your hair, and your skin. No amount of air-freshener or perfume can fully mask this smell.	3
A17	When you quit smoking, you will gain an improved sense of well-being. You can enjoy activities without feeling exhausted. It’s time to think about quitting.	3
A18	Did you know that quitting smoking can give you a whiter smile, fresher breath and clearer, younger looking skin?	3
A19	What is your reason to quit? Valerie, a former smoker, said being physically away from her kids and the noise in her head to get away to smoke bothered her.	2
A20	Michael, a former smoker, thinks it’s important to quit because it helps extend your life with fewer health problems. It also saves money for other things.	2
A21	Smoking can make breathing hard. After you quit you may breathe better and have more energy. Quitting also lowers your risk of getting cancer from smoking.	2
A22	Long term risks of smoking include heart attacks and stroke, cancer, osteoporosis, and long-term disability.	2
A23	COPD is the 4th leading cause of death in the United States. COPD is not fully reversible, but quitting smoking can help you breathe better and feel better.	2
A24	Research shows that quitting smoking at any age can increase your life span by an average of 7 years.	2
A25	Advice from Andrea, a former smoker: Estimate how much you’ve spent on cigarettes daily, weekly, monthly, yearly. How much will it cost you over your lifetime?	1
A26	Darcy, a former smoker, suggests saving all the money you spent on cigarettes as if you were buying them. Use it as a reward!	1
A27	Many health and life insurance companies charge lower premiums to non-smokers.	1
A28	The cost of smoking goes beyond the pack of cigarettes. Smokers have greater health care costs than non-smokers because smoking causes many health problems.	1
A29	If you smoke one pack of cigarettes/day, you are spending more than $200 per month to smoke. What could you do with the money you’d save from quitting smoking?	1
A30	One pack of cigarettes per day for 10 years will cost you nearly $25,000. How much are YOU spending on smoking?	1

Note. Theme codes: 1 = Financial cost/rewards; 2 = Health; 3 = Quality of life; 4 = Challenges of quitting; 5 = Motivations to quit/reasons to quit

Using the MADM [47], the decision matrix for each mood state shows each row accounting for each cessation message and each column accounting for each measure. Messages were ranked for each study (crowdsourcing, psychophysiology) and then ranked overall. Text for the exact message can be found in Table 4.

Finally, we compared ratings of each message between each of the mood categories by subtracting one ranking in a mood category from one in another mood category for the same message. If rankings spanned at least 10 rankings (i.e., one tertile), then the message was categorized as mood sensitive, meaning the message ranked according to mood state.

## Results

### Participants

Participants in the crowdsourcing study were on average 41.13 years old (SD = 11.33), mostly self-identified as female (52.7%), White (82.3%), and Non-Hispanic/Latino (92.9%), received some of a college degree (42.5%), described their health as “good” (42.9%), and their financial life as not difficult at all (27.2%). Participants reported smoking an average of 13.47 cigarettes per day. Due to incomplete data, 33 participants were dropped for analysis resulting in a sample of *N* = 567 [positive (*N* = 184), negative (*N* = 189), neutral (*N* = 194)].

Participants in the psychophysiological study were on average 46.98 years old (SD = 14.71), mostly self-identified as female (58.5%), White (78.0%), Non-Hispanic/Latino (82.9%), received some of a college degree (43.9%), described their health as “good” (53.7%), and their financial life as not difficult at all (36.6%). Participants reported smoking an average of 12.04 cigarettes per day. It should be noted that some messages in each mood condition had as low as only two participants with peak amplitudes and one message in one condition with no peak amplitudes (meaning there was no reaction of arousal during viewing, thus receiving a score of 0). Participants for the significance testing of heart rate and skin conductance with incomplete data (missing data points for more than 3 messages) were dropped in analysis.

Individuals in the psychophysiological study (*M* = 46.98) were slightly older than individuals in the crowdsourcing study (*M* = 41.13; *p* = .017) and rated their financial life as slightly easier (psychophysiological study *M* = 3.90; crowdsourcing study *M* = 3.41; *p* = .01). No other differences between study participants were significant.

### Psychophysiology data significance tests

Participants’ heart rate change scores from baseline were analyzed with a 5 (theme: motivation to quit, challenges in quitting, quality-of-life, health harms, financial costs/rewards × 6 (message per theme) × 14 (time in seconds) repeated measures ANOVA. For positive mood state, there was a marginally significant theme × message × time linear interaction (*F*(1, 10) = 3.48, *p* = .091, η_p_^2^ = 0.26). Specifically, the health themed messages performed the least well and the financial themed messages showed the largest deceleration over the duration of viewing. Cardiac deceleration indicates more cognitive resources allocated to message processing [46]. For negative mood state, the interactions with message theme were not significant. For neutral mood state, there was a significant theme × time quadratic interaction (*F*(1, 8) = 5.14, *p* = .053, η_p_^2^ = 0.39). Cardiac response curves that are quadratic indicate a greater orienting response [46] to a specific theme category. Results showed that the challenges in quitting theme category performed the least well and the health and quality of life had the largest cardiac deceleration points.

Participants’ skin conductance change scores from baseline were analyzed with a 5 (theme: motivation to quit, challenges in quitting, quality-of-life, health harms, financial costs/rewards × 6 (message per theme) × 14 (time in seconds) repeated measures ANOVA. There were no significant associations.

### Self-report data and rankings

Tables 1 and 2, and 3 show the mean scores for the self-reported outcomes in the crowdsourcing study (motivation to quit, message receptivity, and message relevance). In the positive mood condition, the top two ranked messages were in the challenges in quitting message theme (A10: Feelings of stress are normal when quitting smoking. You are not alone! Talk with your doctor or a friend about ways to reduce stress before your quit date; A9: Realize the first 48 h after quitting is the most difficult time. Make a plan to handle it. It gets better! ), and the third was in the quality-of-life theme (A16: The smell of smoke gets into your clothes, your car, your home, your hair, and your skin. No amount of air-freshener or perfume can fully mask this smell).

In the negative mood condition, the top three ranked messages were all in the challenges in quitting theme (A10: Feelings of stress are normal when quitting smoking. You are not alone! Talk with your doctor or a friend about ways to reduce stress before your quit date. A9: Realize the first 48 h after quitting is the most difficult time. Make a plan to handle it. It gets better! A7: Did you know smoking can influence your mood? If you feel lonely or depressed while quitting, talk with your doctor. There is treatment to help).

In the neutral mood condition, the top two ranked messages were in the challenges in quitting theme (A10: see above A7: Did you know smoking can influence your mood? If you feel lonely or depressed while quitting, talk with your doctor. There is treatment to help.) followed by a message in the quality-of-life theme (A16: The smell of smoke gets into your clothes, your car, your home, your hair, and your skin. No amount of air-freshener or perfume can fully mask this smell).

### Psychophysiological data and rankings

Tables 1 and 2, and 3 show the mean scores for the biobehavioral outcomes in the psychophysiology study (heart rate, skin conductance, eye-tracking). The top ranked messages in the positive mood condition were in the financial theme (A30: One pack of cigarettes per day for 10 years will cost you nearly $25,000. How much are YOU spending on smoking? ), followed by another financial theme message (A27: Many health and life insurance companies charge lower premiums to non-smokers.), and motivations to quit theme (A1: Brandon, a current smoker, said that to get focused on quitting you should get psyched about a vacation, and the money you spend on cigs could go to that.).

Top ranked messages in the negative mood condition were in the financial theme (A26: Darcy, a former smoker, suggests saving all the money you spent on cigarettes as if you were buying them. Use it as a reward! ), challenges in quitting (A10: Feelings of stress are normal when quitting smoking. You are not alone! Talk with your doctor or a friend about ways to reduce stress before your quit date.), and quality-of-life theme (A18: Did you know that quitting smoking can give you a whiter smile, fresher breath and clearer, younger looking skin? ).

In the neutral mood condition, top ranked messages were in the challenges in quitting theme (A11: The worst withdrawal symptoms will occur in the first week after quitting, but by one month, most symptoms are gone.), quality-of-life theme (A15: No matter how many years you have been smoking, quitting can increase your life span and give you a better quality of life), and motivations to quit theme (A6: Are you worried about how smoking affects your family and friends? Try to avoid smoking around your loved ones.).

### Integration of self-report and psychophysiology data and rankings

Tables 1 and 2, and 3 show the overall rankings for each message after integrating the self-report and psychophysiological data. For the positive mood condition, three messages ranked in the top spot: challenges in quitting message (A9: Realize the first 48 h after quitting is the most difficult time. Make a plan to handle it. It gets better! ), financial (A30: One pack of cigarettes per day for 10 years will cost you nearly $25,000. How much are YOU spending on smoking? ), and motivations to quit (A1: Brandon, a current smoker, said that to get focused on quitting you should get psyched about a vacation, and the money you spend on cigs could go to that.).

For the negative mood condition, a challenges in quitting message theme ranked top (A10: Feelings of stress are normal when quitting smoking. You are not alone! Talk with your doctor or a friend about ways to reduce stress before your quit date), followed by a quality-of-life message (A16: The smell of smoke gets into your clothes, your car, your home, your hair, and your skin. No amount of air-freshener or perfume can fully mask this smell), and finally, a financial message (A26: Darcy, a former smoker, suggests saving all the money you spent on cigarettes as if you were buying them. Use it as a reward! ).

For the neutral mood condition, the top three ranked messages all were in the challenges in quitting theme (A10: Feelings of stress are normal when quitting smoking. You are not alone! Talk with your doctor or a friend about ways to reduce stress before your quit date.), followed by a quality of life message (A15: No matter how many years you have been smoking, quitting can increase your life span and give you a better quality of life.), and challenges in quitting (A8: There will be challenges to quitting, especially during the first few weeks. Make a list of things you can do, like exercise, to help with these challenges.).

## Discussion

The current study tested CTHC messaging based on the mood state of adults who smoke. Using a MADM integrating self-report and psychophysiological data, we found that the mood state during message exposure impacted how adults who smoke ranked different messages as most and least appealing. In the positive mood condition, the three top ranked messages spanned themes about challenges in quitting, financial cost/rewards, and motivations to quit. In the negative mood condition, the three top ranked messages spanned themes of challenges in quitting, quality-of-life, and financial cost/rewards. Finally, in the neutral condition, all top three ranked messages were in the challenges in quitting and quality of life themes. Information garnered from this study will help inform future CTHC messaging and interventions to tailor messaging based on individuals’ moods.

Comparing these results to the previously published significance testing of the crowdsourcing data [26], the current study found that specific messages in the theme categories of challenges in quitting, financial cost/rewards, and motivations to quit may be best suited for those in a positive mood state. Our crowdsourcing data generally found that the financial, health, quality of life, and challenges of quitting themes were associated with increased motivations to quit when in a positive mood state compared to those in a negative mood state [26]. The data in this study further drill down on which specific messages may be most effective while integrating various psychophysiological measures.

For those in the positive mood condition, the top ranked messages focused on acknowledging how hard the first 48 h of quitting is, how much money could be saved by quitting, and getting excited about vacations that one could go on with the money saved. The lowest ranked messages focused on the loss of years of life due to smoking, loss of time with children, and the triggers of smoking. These results might best be explained through Mood Management Theory [63, 64]. Borrowed from the media psychology literature, mood management theory posits that individuals enjoy and choose content (e.g., entertainment, messaging, advertising) that *manages* their current mood state [63, 64]. For instance, individuals have the motivation (either consciously or unconsciously) to optimize their current mood state; positive mood states want to be maintained, negative states want to be improved; anxious feelings beg for calm; and dull feelings choose for excitement. In the current study, best performing messages focused on maintenance of the positive state that include messaging emphasizing empathy for how hard quitting is and how much money can be saved and what it can be spent on. The worst performing messages seem to touch on aspects that could turn the positive state to a negative one – such as the real consequences of smoking including loss of time. Additionally, past research has shown that individuals who smoke have increased attention and are more likely to systematically process information when shown smoking cessation information when in a positive mood state [25]. Our research furthers this in presenting specific messages that may further increase smoking cessation when individuals are in a positive mood state.

Those in the negative mood condition responded best to messages about feelings of stress are normal, the inability to get rid of the smoke smell, and rewarding oneself with the money that could be saved from quitting. Interestingly, the worst performing message was about how cigarettes took a person away from their children, how much money cigarettes cost over a lifetime, and how quitting lowers the risk of lung cancer. The better performing messages emphasized empathy and gains from quitting, while the worst performing emphasized things to be lost from continuing to smoke. In fact, some research has shown that individuals are more persuaded by gain-framed messages (vs. loss-framed) for prevention behaviors [65], such as quitting smoking. In this study’s rankings, better performing messages emphasized a gain frame. However, this finding should be taken with caution, as other studies, that did not measure mood state, found that loss-framed messages may encourage individuals to virtually engage in an online smoking cessation intervention [66]. Future work should explore this association. Other poorly performing messages emphasized the long term health risks of smoking – essentially bringing up hard truths and not managing the negative feelings as mood management theory would predict [63, 64]. Furthermore, prior research suggests that individuals in a negative mood may use more effortful processing [29], so the messages in this study that performed well may better be stored in memory for those in a negative mood state.

Those in the neutral condition preferred messages in the challenges of quitting theme. These messages emphasized compassion and tips in quitting. The worst performing message was also in the challenges in quitting theme which asked about triggers to smoke, while the many of the other poorly performing messages were in the financial cost/rewards theme. These findings may indicate that those in a neutral mood state might not react advantageously to financial themed messages.

Finally, the heart rate significance testing revealed that those in the positive mood condition had less cognitive resources allocated to processing the health themed messages, while the messages about financial costs and rewards had the largest deceleration point and cognitive resources allocated to message processing. Similar to the aggregated results from the MADM, Mood Management Theory [63, 64] may partially explain this as health consequences of smoking may be bring on a less positive state and financial messages may be an easier theme to process while maintaining a positive mood state. Heart rate data significance testing also indicated that those in the neutral mood condition had less cognitive resources allocated to processing messages about the challenges in quitting messages, while the messages about health and quality of life had the most cognitive resources allocated to message processing. When in a neutral mood state, individuals may be more willing to processing messaging about health and quality of life. Neutral mood states may convey feelings of safety and security, thus diminishing the perception of potential threats [29]. 

Whereas this study provided important insights into CTHC messaging dependent on mood, it has limitations. First, the crowdsourcing study had more stringent inclusion criteria for smoking status; thus, the two studies could have included participants of different smoking statuses (i.e., heavy vs. light). Second, the mood manipulation check was conducted using validated pictures, however, moods may be more intense or less intense in the real-world affecting responses to smoking cessation messages. Third, the crowdsourcing study included eligible individuals from all over the United States, while the in-lab study included those close to a large northeastern university. Thus, location may affect the outcomes.

To our knowledge, this study was among the first to test message responses in conjunction with mood states of adults who smoke. Results showed that those in different mood states responded differently to messages in different themes. For smoking cessation messaging, those in a positive mood may respond best to messages that bolster their positive state and without including negative triggers. Those in a negative mood state may prefer to focus on gains to quitting smoking. Those in a neutral state may prefer messaging focused on empathy and actionable steps. These insights should be further tested in a real-world setting and should be examined for behavior change in adults who smoke. However, this study is the first step in integrating mood into CTHC messaging interventions for smoking cessation.

## Data Availability

The data underlying this article will be shared on reasonable request to the corresponding author.
